# Simulated Micro-, Lunar, and Martian Gravities on Earth—Effects on *Escherichia coli* Growth, Phenotype, and Sensitivity to Antibiotics

**DOI:** 10.3390/life12091399

**Published:** 2022-09-08

**Authors:** Lily A. Allen, Amir H. Kalani, Frederico Estante, Aaron J. Rosengren, Louis Stodieck, David Klaus, Luis Zea

**Affiliations:** 1 BioServe Space Technologies, University of Colorado Boulder, Boulder, CO 80309, USA; 2 Smead Aerospace Engineering Sciences Department, University of Colorado Boulder, Boulder, CO 80309, USA; 3 Molecular, Cellular and Developmental Biology Department, University of Colorado Boulder, Boulder, CO 80309, USA; 4 Department of Mechanical and Aerospace Engineering, University of California San Diego, San Diego, CA 92093, USA

**Keywords:** clinostat, minimum inhibitory concentration, MIC, rpoS, cell size, aggregation, ciprofloxacin, gentamicin, urinary tract infection, UTI, spaceflight

## Abstract

Bacterial behavior has been studied under microgravity conditions, but very little is known about it under lunar and Martian gravitational regimes. An Earth-based approach was designed and implemented using inclined clinostats and an in-house-developed code to determine the optimal clinorotation angular speed for bacterial liquid cultures of 5 RPM. With this setup, growth dynamics, phenotypic changes, and sensitivity to antibiotics (minimum inhibitory concentration (MIC) of two different classes of antibiotics) for three *Escherichia coli* strains (including uropathogenic) were examined under simulated micro-, lunar, and Martian gravities. The results included increased growth under simulated micro- and lunar gravities for some strains, and higher concentrations of antibiotics needed under simulated lunar gravity with respect to simulated micro- and Martian gravities. Clinostat-produced results can be considered suggestive but not determinative of what might be expected in altered gravity, as there is still a need to systematically verify these simulation devices’ ability to accurately replicate phenomena observed in space. Nevertheless, this approach serves as a baseline to start interrogating key cellular and molecular aspects relevant to microbial processes on the lunar and Martian surfaces.

## 1. Introduction

Humans carry roughly as many bacterial cells in and on our bodies as we have total mammalian cells [1]. By the nature of this, we will ubiquitously share space habitats with bacteria. Hence, it is important to understand how an altered gravitational environment may affect bacterial behavior and, in the context of human health concerns, the efficacy of antibiotics. Although no overarching definitive conclusions have yet been reached [2,3], many studies performed in space have shown altered bacterial behavior in microgravity with respect to 1 g on Earth [4], including modified cell and biofilm phenotype [5,6,7,8], increased virulence [9], and reduced bacterial susceptibility to antibiotics [7,10,11,12,13,14,15,16,17,18,19,20,21,22].

While these studies have interrogated bacterial behavior and its potential consequences for crew health in the microgravity environment of orbital flight, very little is known on these topics under lunar and Martian gravitational regimes, 1.62 m/s^2^ (~⅙ g) and 3.72 m/s^2^ (~⅜ g), respectively. Of particular interest are processes related to urinary tract infections (UTI) given that urosepsis (which can occur if a UTI is not treated properly) is the third most likely reason for emergent medical evacuation from the International Space Station (ISS) [23]. *Escherichia coli* is the most commonly isolated UTI pathogen [24], and is also typically used as a bacterial model organism for spaceflight research [19]. As part of a NASA-funded study using ten microbes (results of other species to be published separately), this manuscript describes experiments focused on characterizing growth dynamics (lag phase duration, exponential growth rate, and final cell counts at stationary phase), phenotypic changes (cell diameter and length and aggregation), and the sensitivity to antibiotics (minimum inhibitory concentration (MIC) of two different classes of antibiotics) for three *E. coli* strains cultured under simulated micro-, lunar, and Martian gravities. Our methodology to simulate some aspects of these gravitational conditions—inclined clinostats and a code to determine an optimal clinorotation angular speed for bacterial liquid cultures—are also explained.

## 2. Background

**Figure 1:**
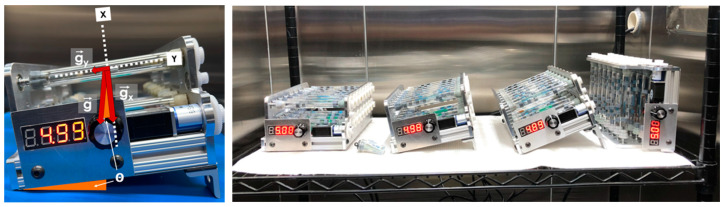
**Left**: BioServe’s FPA Clinostat inclined at an angle theta. While the x component of the gravity vector on any given cell is randomized via clinorotation, the y component replicates reduced sedimentation and buoyancy as would occur at a gravitational level between microgravity and 1 g. **Right**: All four clinostats for each gravitational regime configured for experimentation inside BioServe’s environmental test chamber.

**Figure 2:**
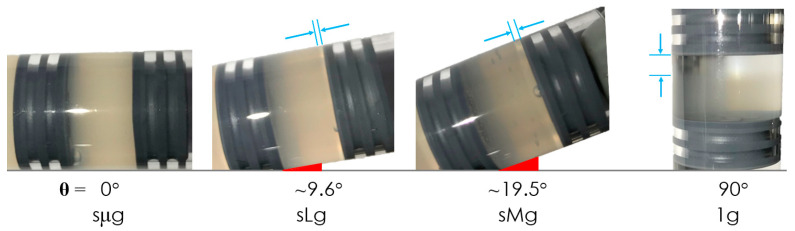
Differences in non-motile bacterial sedimentation rates can be observed between FPAs clinorotated at different angles at 24 h of growth; sµg, sLg, and sMg refer to simulated micro-, lunar, and Martian gravities, respectively. Shown here are cultures from a study reported separately using non-motile *Salmonella* are shown, as no images were taken of this particular study. Cellular sedimentation (indicated by cyan lines) can be observed being collinear to the axis of rotation, regardless of the angle of clinostat inclination.

Although some mechanistic observations have been made [19,20,22], it is not yet clearly elucidated how biophysical and molecular genetic phenomena—such as the activation of antibiotic resistant pathways—change within gravitational regimes between microgravity and 1 g, or how these environments impact bacterial phenotype, physiology, or behavior. To further interrogate the effects of reduced gravities, lunar and Martian gravitational regimes were simulated on Earth to the extent possible, using BioServe’s in-house developed clinostats. While rotating wall vessels and clinostats allows for the simulation of some aspects of the microgravity environment (also called ‘low-shear modeled microgravity’), the replication of regimes between microgravity and 1 g requires an additional step. Clinostats continuously rotate a container fully loaded with a liquid culture at a constant speed with the goal of maintaining cells within a determined depletion zone [25,26]. To ensure the rotation is not so fast that cells are centrifuged away from this zone, or too slow so that they would sediment out of suspension, a code was developed to determine the angular velocity to be used based on parameters such as cell length, diameter, mass, buoyancy-corrected mass, density, medium density and viscosity, and vessel diameter via a set of second order, linear, separable, non-homogeneous stiff differential equations [27]. These ‘FPA Clinostats’ could be set horizontally (to simulate microgravity), vertically (for 1 g controls), or at an angle in between to replicate certain aspects of regimes between micro- and 1 g (see Figure 1 and Figure 2) [28,29,30,31,32,33]. It is acknowledged that, just as clinostat-produced environments are not the same as actual microgravity (they cannot remove Earth’s gravity and can introduce confounding factors), inclined clinostats are not the same as the actual lunar or Martian surface. Nevertheless, they serve as a reasonable baseline to start interrogating key cellular and molecular aspects relevant to microbial processes on the lunar and Martian surfaces.

The *E. coli* strains chosen for this study were (i) ATCC^®^ 4157™, previously flown by our group on eight space shuttle flights aboard STS-37, -43, -50, -54, -57, -60, -62, and -95 [28,34] and to the ISS [19], (ii) AMG1, a uropathogenic clinical isolate from the Stanford Medical Center [35], and (iii) an *rpoS* knockout mutant of AMG1 to study this regulator gene’s pathways (associated with resistance to multiple types of stresses and resistance to some antibiotics on Earth via its product, the σ^S^ sigma factor [36,37,38,39,40]. The latter two strains were flown to space on NASA’s EcAMSat [40,41,42]. The uropathogens were challenged with Ciprofloxacin, the most commonly prescribed drug for UTI [43]. ATCC^®^ 4157™ was challenged with Gentamicin sulfate as in [19].

## 3. Materials and Methods

### 3.1. Setup

*E. coli* AMG1 and its mutant strain were cultured in modified artificial urine medium supplemented with glucose and a high concentration of phosphate (mAUMg hi-Pi) [5,44] to replicate human urine. While glucose concentrations in human urine are usually 0 to 0.8 mM or lower [45], our growth medium had a higher concentration, 2 mM, for comparison against our group’s other spaceflight studies (to be published separately) and to ensure bacterial growth would take place, enabling us to interrogate the role of simulated gravitational conditions. *E. coli* 4157 was cultured in LB lennox broth (Sigma Aldrich, St. Louis, MO, USA, L3022). All strains were grown at 37 °C. Cultures were housed in BioServe’s FPAs, enclosed by rubber septa, and then loaded into BioServe’s FPA clinostats (Figure 1). Washing, sigmacoting^®^, and sterilization was performed on all hardware prior to experimentation, per Zea et al. [20].

### 3.2. Bacterial Motility

**Figure 3:**
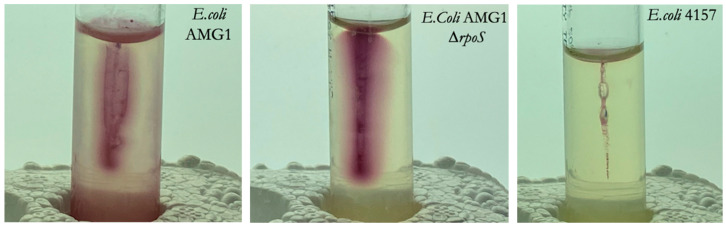
AMG1 was semi motile, AMG1 ∆*rpoS* was motile, and ATCC 4157 was non-motile.

To determine the motility of each strain, a needle dipped in bacterial culture was inserted into motility tubes (Thermo Scientific, Waltham, MA, USA, Cat. No. R061410) and was incubated at 37 °C for 24 h. A motile culture was indicated by a dispersion of pink color resulting from the bacterial reduction of Tetrazolium (TTC), a metabolite within the agar (Figure 3). The motility tests showed that *E. coli* AMG1 was semi motile, *E. coli* AMG1 ∆*rpoS* was motile, and *E. coli* ATCC 4157 was non-motile.

### 3.3. Experimental Approach

A 24-h culture was diluted by 1:100 and loaded into the FPAs. The optical density (OD)_595_ at the start of the experiment was 0.002 on average. The FPA consisted of four independent chambers, each containing 1.3 mL of culture, segregated by rubber septa. One FPA was sacrificed at each timestamp for OD data acquisition (the last timestamp had eight replicates for improved statistics). Timestamps were determined by preliminary growth curve experiments conducted at 1 g, and in the case of the *E. coli* AMG1 and *E. coli* AMG1 ∆*rpoS* strains, were based on the timeline presented in Pagden et al. [40]. Additional FPAs were loaded with 10 mL of culture each for supplemental analyses, including microscopy.

For the antibiotic sensitivity experiments, each FPA had two independent chambers, each containing 3 mL of culture, and enough FPAs were loaded so that there were four replicates for each tested antibiotic concentration, per each gravitational condition. The desired antibiotic concentration was prepared with the same media used in the bacterial culture as diluent and was filter sterilized. In each test, four concentrations were assessed. Drugs (Ciprofloxacin (Fisher Sci., Waltham, MA, USA, Cat. No. AC456880050) or Gentamicin Sulfate (Fisher Sci., Waltham, MA, USA, Cat. No. BP918-1)) were introduced in the bacterial culture when they reached exponential phase. At this time, two FPAs per gravitational regime were sacrificed for OD data acquisition to quantify cell concentration at the time of antibiotic introduction. Then, the four antibiotic concentrations were introduced to their respective FPAs by injecting 1 mL of antibiotic into each chamber. All FPAs were returned to their FPA Clinostat and environmental test chamber at 37 °C for 12 additional hours, following the MIC protocol described by Andrews [46], adapted to be implementable in this spaceflight hardware. In this assay, bacterial cultures are challenged with increasing concentrations of antibiotics to find the minimum concentration of a given drug sufficient to inhibit bacterial growth under those conditions. Growth inhibition is determined by statistically comparing cell concentration after 12 h of drug challenge against the cell concentration at the time of antibiotic introduction.

### 3.4. Code to Determine Clinostat Rotational Speed

One of the unique attributes of microgravity at the cellular level in a liquid medium is an altered extracellular mass transport profile. On Earth, a cell in a liquid medium will be subject to gravity-driven forces and resultant flows, including sedimentation, buoyancy, and convection, which help transport molecules from one location to another. In microgravity, however, and for non-motile cells in particular, mass transport is limited to diffusion arising from Brownian motion, surface effects, and/or gradient-driven transport phenomena, translating into a quiescent, quasi-stable extracellular environment where incoming substrate is thought to become limited, and excreted metabolic byproducts accumulate around a cell [20,33]. Some aspects of this quiescent environment can be replicated using a clinostat on Earth to continuously rotate a vessel filled with a liquid culture, with the caveat that the 1 g vector is never removed as it is operated on Earth. Thus, the suspended cells still experience their full weight. The angular speed (*ω*) at which this vessel is rotated is a function of cellular and medium properties, including but not limited to cell length and diameter (normalized into an equivalent Stoke’s radius (*a*), mass (*m*), buoyancy-corrected mass (m*), particle density ($\rho_{p}$), medium density ($\rho_{f}$), viscosity (*ν*), and Stoke’s drag constant (*f*), as well as the vessel diameter (*d*). If the vessel is rotated too slowly, the cell will sediment away from the quiescent zone; if it is rotated too quickly, however, the cell will be centrifuged out of the quiescent zone. The position of a particle at a given time (*t*) can be modeled based on the aforementioned parameters via two second order, linear, separable, non-homogenous stiff differential equations [25,27,47]:

(1) $$\ddot{x}+\frac{f}{m}\dot{x}-\frac{m^{*}}{m}\omega^{2}x=-\frac{m^{*}g}{m}\sin\omega t$$

(2) $$\ddot{y}+\frac{f}{m}\dot{y}-\frac{m^{*}}{m}\omega^{2}y=-\frac{m^{*}g}{m}\cos\omega t$$

where *x* and *y* refer to the axes of a coordinate system upon which the vessel’s circular cross section is centered at the (0,0) position.

We developed a MATLAB^®^ code based on the ode15 s ordinary differential equation solver [27], to determine the longest duration a cell would stay within the putative quiescent zone under these conditions as a function of angular speed (accessible here: https://github.com/SpaceLuisZea/Clinostat) (accessed on 9 August 2022). As inputs to the code, we used a bacterial Stoke’s diameter of $5.6\times 10^{-5}\;\mathrm{cm}$, cell mass of $4.0\times 10^{-13}\;g$, growth medium viscosity of 0.0102 g/cm·s and density of $1.011\;g/{\mathrm{cm}}^{3}$ (for reference, water’s viscosity at 25 °C and density are 0.0091 g/cm·s and $1.000\;g/{\mathrm{cm}}^{3}$, respectively), and an arbitrary quiescent zone diameter of 10 µm. This information (as well as recommended rotational speed) has been shared with other groups who have used our clinostats and FPAs [48].

### 3.5. Data Acquisition

Cultures were extracted and transferred into a 24-well plate for mixing, and from there aliquots were transferred to a 96-well plate. OD was measured at 595 nm for the growth curve and at 600 nm for MIC experiments. Cultures from the last timestamp of the growth curve for each regime were preserved by mixing 1 mL of 4% PFA (Fisher Scientific, Waltham, MA, USA, Cat. No. AAJ61899AK) and 1 mL of sample. For microscopy, samples were centrifuged for two to three minutes at 900 g’s and an aliquot was transferred to a slide. Microscopy was performed on an OLYMPUS.IX81 Inverted Widefield Microscope with a 100X 1.40NA SAPO objective lens and images were captured via a Hamamatsu Orca R2 CCD camera.

### 3.6. Statistical Analysis

At each timestamp and for each gravitational regime, four replicates were sacrificed to acquire OD data. For each replicate, four aliquots were assessed, hence the OD for each regime at each timestamp was the mean of the averages of four aliquots per each of the four replicates. To determine if there were statistical differences between the gravitational regimes at 24 h, an Analysis of Variance (ANOVA) for parametric datasets or Dunn test for nonparametric data sets was performed. Slopes between the beginning and end of the exponential phase were found using Microsoft Excel’s “slope” function. These slopes were compared to the 1 g control (slope_gravitational regime_/slope_1 g_). R^2^ values were found by using Excel’s RSQ function. This same process was used to calculate the “death rate” via the slope between the stationary phase and the last timepoint in the experiment. The average length and width were calculated for 50 measurements of each condition, preserved at the last timestamp. For MIC tests, a Tukey, Kruskal, or Dunn test was performed between OD at the time of antibiotic introduction and after 12 h of being challenged with antibiotics. The lowest concentration for which there was no significance with respect to the OD at time of drug introduction was the MIC.

## 4. Results

### 4.1. Calculated Time to Depart a Putative 10 µm Depletion Zone

**Figure 4:**
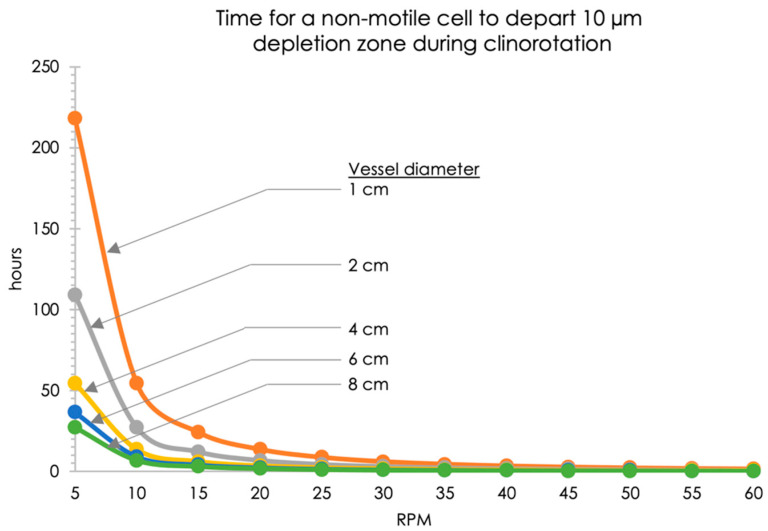
Estimated time needed for a non-motile cell to depart an arbitrary 10 µm depletion zone as a function of clinostat angular velocity (5–60 rpm) and vessel diameter (1–8 cm), per our MATLAB^®^ code.

We used our MATLAB^®^ code to simulate and subsequently determine how long it would take a non-motile bacterial cell to leave a depletion zone of 10 µm in diameter as a function of rotational speed and vessel diameter. Figure 4 shows the code output in this regard, indicating that for an FPA-like vessel (1 cm in diameter) and 5 RPM rotational speed, a non-motile bacterial cell could be expected to remain within this depletion zone (i.e., it would not sediment or be centrifuged away from it) for ~220 h, or just over nine days. Given that this was longer than our expected two-day long test, this RPM was chosen.

### 4.2. AMG1 and ∆rpoS Results

#### 4.2.1. Growth Dynamics

**Figure 5:**
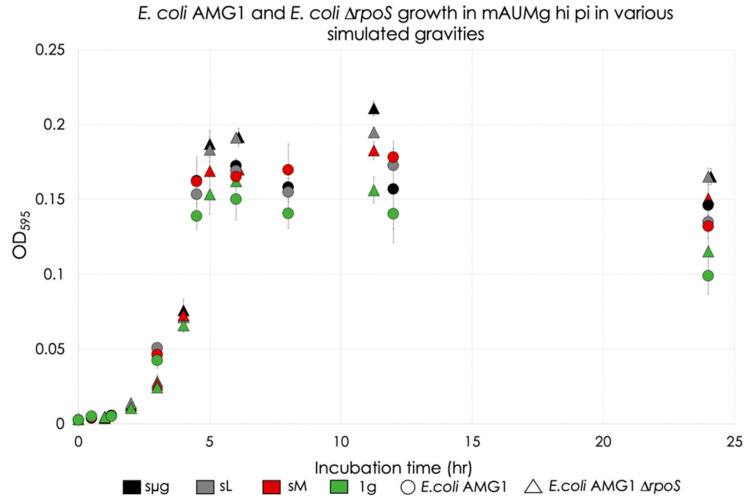
*E. coli* AMG1 and *E. coli* AMG1 ∆*rpoS* growth in mAUMg-high Pi in various simulated gravities. Circles indicate *E. coli* AMG1, and triangles indicate the mutant strain. Error bars are standard error with a 95% confidence interval for this figure and all other data figures (*n* = 4 for all data points except the last timestamp, which has *n* = 8). Timestamps 6 and 24 h of the *E. coli* AMG1 ∆*rpoS* dataset were moved to the right for clarity.

Figure 5 shows the growth dynamics of *E. coli* AMG1 and *E. coli* AMG1 ∆*rpoS* under various simulated gravities. Limited by the times of data collection, the OD data indicate that the lag phase lasted to at least 1.25 h and 1 h for *E. coli* AMG1 and AMG1 ∆*rpoS*, respectively. The beginning and end of the exponential growth phase were 1.25 and 4.5 h for *E. coli* AMG1, and 3 and 5 h for AMG1 ∆*rpoS*, respectively. Slopes between these points are summarized in Table 1.

For *E. coli* AMG1, the stationary phase was reached at 6 h for all regimes. At the 6 h timepoint, significance was only observed between simulated microgravity and the 1 g control (13% increase under simulated microgravity with respect to 1 g, *p* = 0.036). To maintain consistency between the strains, statistical analysis was performed at the 12 h timepoint as well. No significant differences were observed between any regimes for *E. coli* AMG1.

**Figure 6:**
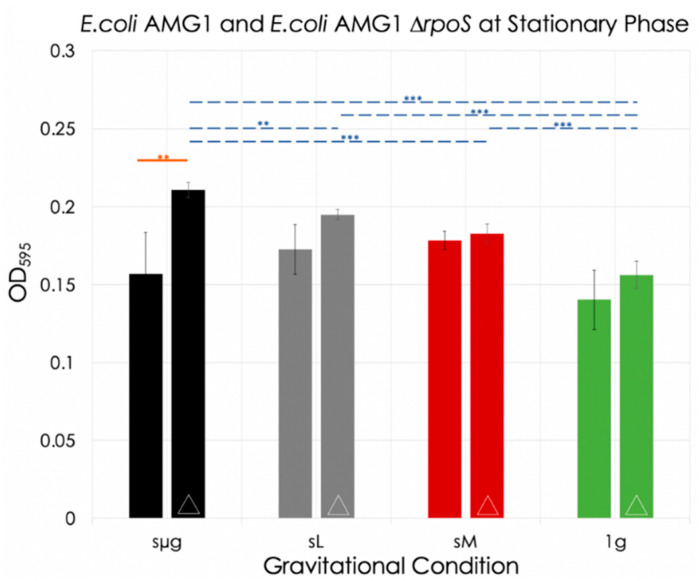
*E. coli* AMG1 and *E. coli* AMG1 ∆*rpoS* ODs at 12 h and 11.25 h (respectively) for each gravitational regime. Bars with the symbol ‘∆’ are data for the mutant strain. The blue dotted lines indicate significance between regimes for *E. coli* AMG1 ∆*rpoS*, and the orange line indicates significance between strains for a given gravitational regime (no significance was observed for *E. coli* AMG1 between regimes). **: 0.001 < *p* ≤ 0.01; ***: *p* ≤ 0.001.

For *E. coli* AMG1 ∆rpoS, cultures grown under simulated lunar gravity and at 1 g reached the stationary phase at 6 h. Under simulated micro and Martian gravities, growth continued until at least 11.25 h. Statistical analysis was performed on data from 11.25 h and significant increases in OD were observed in simulated microgravity with respect to the other three gravitational conditions: simulated lunar (8% increase, *p* = 0.0099), simulated Martian gravity (15% increase, *p* < 0.001), and 1 g (35% increase, *p* < 0.001). When compared to the 1 g control, simulated lunar gravity exhibited significant differences (25% increase, *p* < 0.001), but not when compared to simulated Martian gravity. Finally, simulated Martian gravity exhibited significant differences when compared to the 1 g control (17% increase, *p* < 0.001). Comparison of OD between the strains under each gravitational condition shows significance only for the simulated microgravity regime (34% increase of AMG1 ∆*rpoS* with respect to AMG1, *p* = 0.003) (orange line in Figure 6).

The death phase of *E. coli* AMG1 and AMG1 ∆*rpoS* occurred somewhere between 12 and 24 h, and between 11.25 and 24 h, respectively, and is limited by the times of OD data collection of the experiment. Death rate slopes were calculated between these last two timepoints for each strain. This information is summarized in Table 2.

**Figure 7:**
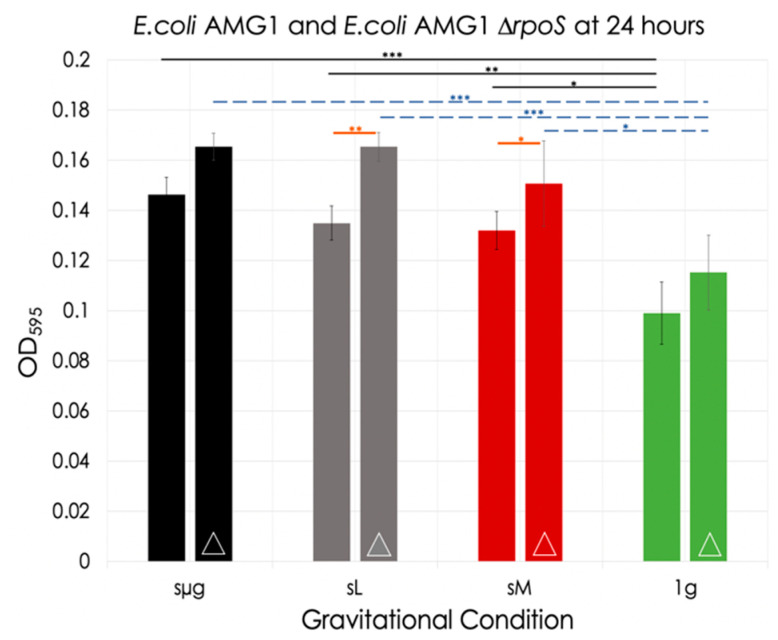
*E. coli* AMG1 and *E. coli* AMG1 ∆*rpoS* optical densities at 24 h for each gravitational regime. Bars with the symbol ‘∆’ are data for the mutant strain. The black lines indicate significance between regimes for *E. coli* AMG1, the blue dotted lines indicate significance between regimes for *E. coli* AMG1 ∆*rpoS*, and the orange lines indicate significance between strains for a given gravitational regime *: 0.01 < *p* ≤ 0.05; **: 0.001 < *p* ≤ 0.01; ***: *p* ≤ 0.001.

At the 24 h timestamp, for the wild-type strain, a significant increase in OD was observed under simulated microgravity with respect to the 1 g control (48% increase, *p* < 0.001). Similarly, simulated lunar and Martian gravity showed significant increases when compared to the 1 g control (36% increase, *p* = 0.0037; 33% increase, *p* = 0.0132, respectively). No significance was observed when comparing simulated lunar to simulated Martian gravity. For the mutant strain, while no significant differences in OD were observed between the three reduced gravitational conditions at 24 h, each of them did show statistical differences with respect to the 1 g control. Simulated microgravity had a 44% increase (*p* < 0.001), simulated lunar gravity also had a 44% increase (*p* < 0.001), and simulated Martian gravity had a 31% increase (*p* < 0.001). Comparison between strains under each gravitational condition showed significantly higher ODs for the mutant with respect to AMG1 under simulated lunar (23% increase, *p* = 0.003) and under simulated Martian gravities (12% increase, *p* = 0.045) (orange lines in Figure 7).

#### 4.2.2. Cell Size and Aggregation

Length and width were measured for both strains at the last timestamp, 24 h. Table 3 indicates average cell length and width for each gravitational regime and strain, and how each compares to the 1 g controls. The simulated microgravity regime for the wild-type had the biggest size difference for both length (17% greater) and width (11% greater) compared to the 1 g control. The Dunn test was used to identify statistically significant differences in cell length and width as a function of gravitational regime and strain.

Observations were made for cellular aggregation at the last timestamp. For both strains, no aggregation was observed. Settling however did occur most of all in the 1 g control (FPAs oriented upright) and no settling was observed in simulated microgravity (FPAs oriented horizontally).

#### 4.2.3. MIC of Ciprofloxacin

*E. coli* AMG1 and *E. coli* AMG1 ∆*rpoS* were challenged, separately, with increasing concentrations of Ciprofloxacin (0, 0.125, 0.25, and 0.50 mg/L) to determine the minimum concentration of antibiotic needed to inhibit their growth under each gravitational condition. The MIC was defined as the lowest concentration of antibiotic under which no statistical difference in OD_600_ (via Kruskal and Dunn tests) was observed between the time of drug introduction and 12 h later.

As summarized in Table 4, lower concentrations of Ciprofloxacin were needed to inhibit *E. coli* AMG1 growth under the simulated reduced gravities with respect to the 1 g control, which continued growing under the highest concentration tested. It is noteworthy that the simulated microgravity and simulated Martian gravity conditions required half of what was needed under simulated lunar gravity to inhibit *E. coli* AMG1′s growth. Similarly, *E. coli* AMG1 ∆*rpoS* growth was inhibited under simulated microgravity at a 0.50 mg/L concentration—a concentration that was not inhibitory under the other four tested gravitational conditions.

### 4.3. E. coli 4157

#### 4.3.1. Growth Dynamics

**Figure 8:**
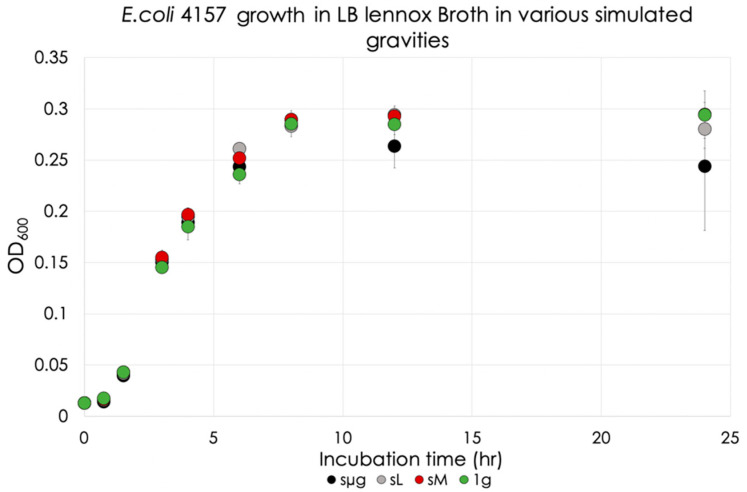
*E. coli* 4157 growth in LB Lennox broth in various simulated gravities.

*E. coli* 4157 was cultured over the course of 24 h as shown in Figure 8. The data indicated that the lag phase lasted 0.75 h for all simulated gravities. Exponential growth slopes were calculated between t = 1.5 and 6 h, and were compared to the 1 g control, as summarized in Table 5. The rate of growth during the exponential phase for each regime showed faster growth under the simulated reduced gravities with respect to 1 g, with no specific pattern: simulated lunar gravity had the highest growth rate, 14% greater than the 1 g control, followed by simulated Martian and microgravity (8 and 5% greater than the 1 g control, respectively).

**Figure 9:**
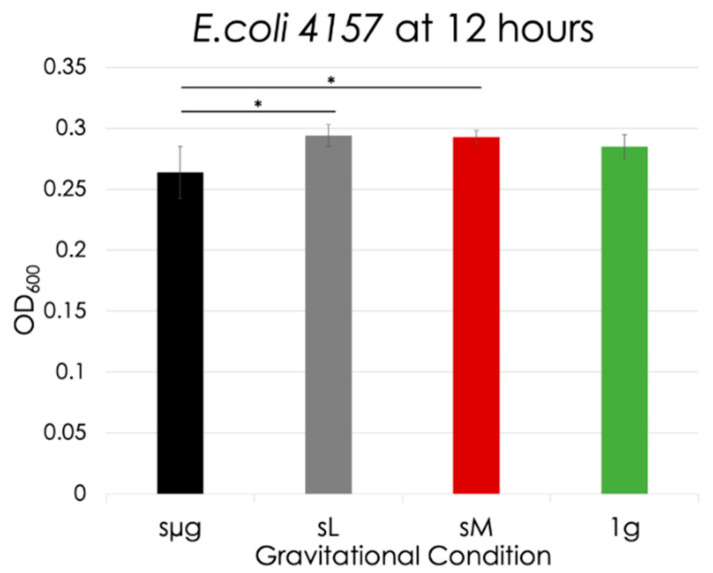
*E. coli* 4157 optical densities at 12 h for each gravitational regime. The black lines indicate significance between regimes. The meaning of the asterisks are as follows: * 0.01 < *p* < 0.05.

The stationary phase was reached at 12 h, and Figure 9 shows the OD of each regime at this time. No significance was observed between the simulated gravities and the 1 g control. Simulated microgravity exhibited significant differences when compared to the other two simulated regimes (simulated lunar gravity: 10% decrease, *p* = 0.029; simulated Martian gravity: 10% decrease, *p* = 0.025). Simulated Martian and lunar gravity were not significant when compared to each other.

**Figure 10:**
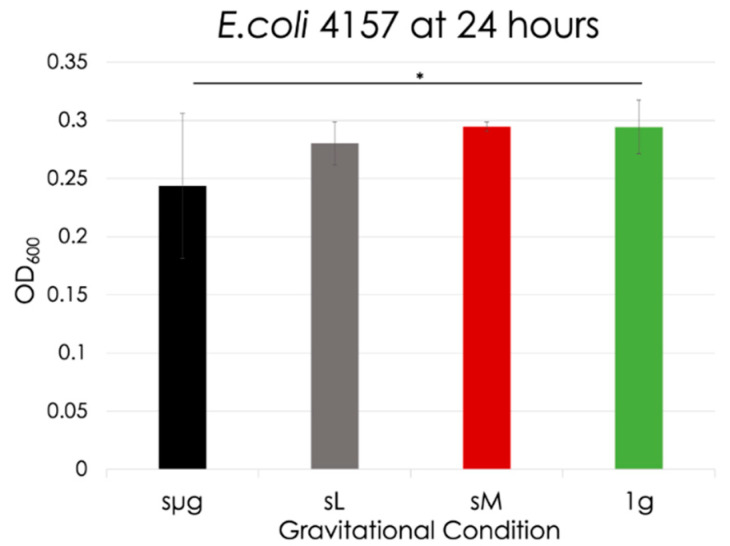
*E. coli* optical densities at 24 h for each gravitational regime. The black line indicates significance between regimes (in this case, significance only occurred between simulated microgravity and the 1 g control). *: 0.01 < *p* < 0.05.

At 24 h, significance was only observed between simulated microgravity and the 1 g control, as shown in Figure 10 (17% decrease with respect to 1 g, *p* = 0.041). No significance was observed when comparisons were made between simulated reduced gravitational regimes. No death curves were observed during the 24 h of this analysis.

#### 4.3.2. Cell Size and Aggregation

**Figure 11:**
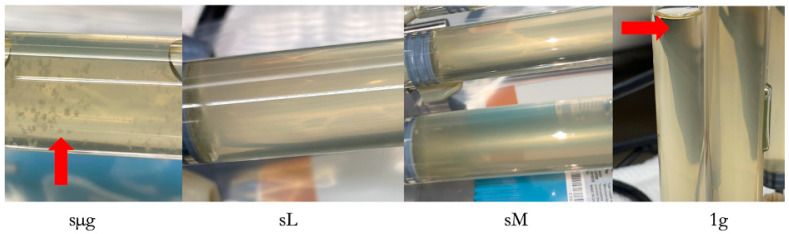
Samples are from the 24 h timepoint for each gravitational regime. Red arrows indicate cellular aggregation (simulated microgravity) or settling (1 g control).

No statistical differences were observed in *E. coli* 4157 cell length or diameter as a function of gravitational condition. In contrast to the AMG1 strains, *E. coli* 4157 aggregation was observed in the simulated microgravity regime. No visible aggregation was present in simulated lunar and Martian gravity. The 1 g control also had no aggregation, but settling was present (Figure 11).

#### 4.3.3. MIC of Gentamicin Sulfate

*E. coli* 4157 was challenged with increasing concentrations (0, 6, 12, 18 mg/L) of Gentamicin Sulfate to determine the MIC. Simulated microgravity, simulated Martian gravity, and the 1 g controls required a 12 mg/L concentration of Gentamicin Sulfate to inhibit *E. coli* 4157 growth. However, the simulated lunar gravity cultures required a higher concentration for growth to be inhibited: 18 mg/L, as summarized in Table 6.

## 5. Discussion

A clinostat may replicate certain aspects of the quiescent extracellular environment experienced in actual microgravity, but it is not the same as microgravity, as it operates on Earth, and thus weightlessness is not achieved. Furthermore, there are key aspects that need to be taken into account when designing reduced gravity experiments on Earth using simulation devices such as clinostats and rotating wall vessels. Namely, the importance of (i) the appropriate angular speed to minimize centrifugation and sedimentation of cells out of their proposed extracellular depletion zone, and of (ii) the use of the smallest diameter vessel possible. Our code indicated that clinorotating a 1 cm diameter vessel at 5 RPM would allow for bacteria to stay within a 10 µm depletion zone for about a week, more than sufficient for our two-day experiment. Given that centrifugal forces are dependent upon the distance between a cell and the axis of rotation, there is an advantage to having the smallest diameter culture vessel as possible, as a cell farther away from this axis will be centrifuged away from its depletion zone faster, hence limiting the duration of the clinostat experiment. In terms of the inclination of clinostats to simulate some aspects of gravity levels between micro and 1 g, the collinearity between cellular sedimentation and the axis of rotation, regardless of the angle of inclination, is a visual indication that this approach achieves the goal of effective axial g-vector alignment (see Figure 2). While our code has its limitations, namely that it does not include Brownian motion or cellular motility when applicable, nor does it take into account cellular aggregation, which is initially unknown, it serves as a first-order analysis to determine the best angular speed based on cellular and media parameters. We recommend that future iterations of this code—which is accessible here: https://github.com/SpaceLuisZea/Clinostat (accessed on 9 August 2022)—include Brownian motion, and that Monte Carlo simulations be run to obtain further numerical results.

More importantly, it is acknowledged that a systematic verification of the validity of clinostats (and rotating wall vessels like HARV, and of random positioning machines, for that matter) needs to be performed by either including clinostat sets in spaceflight experiments, or flying experiments with clinorotated Earth controls (varying angular velocity, for example) with this very specific goal. Otherwise, simulated reduced gravity research performed in any of these types of simulation devices needs to be considered exploratory in nature and remains suggestive of what may be expected in microgravity, not determinative or conclusive in this regard, which also applies to this study.

The growth dynamics results of our experiments indicate there is a direct correlation between motility and lag phase duration: the non-motile strain (4157) took less time to leave the lag phase (0.75 h), the semi motile strain (AMG1) took 1 h, and the motile strain (∆*rpoS*) took the longest (1.25 h). This correlation supports the theory that non-motile cells, in a quiescent extracellular environment like the one experienced in microgravity, are exposed to a relatively high concentration of their own byproducts and secondary metabolites [19,20,22], which may include quorum sensing molecules instructing the culture to begin exponential growth [4]. Comparing these results with those from spaceflight show that our observed reduced lag phase of *E. coli* 4157 matches our previous studies performed on the Space Shuttle [11,34,49]. This correlation may also be non-conclusively interpreted as an indication that the use of clinostats (specifically at 5 RPM and 1 cm diameter vessel), may indeed replicate certain aspects of microbial response in true microgravity. An important caveat to this comparative analysis is that the AMG1 and ∆*rpoS* strains were cultured in minimal media, whereas 4157 was in rich medium.

Analyses of exponential growth rates showed that the AMG1 and ∆*rpoS* strains grew faster under simulated microgravity (17–23% increase with respect to 1 g); simulated lunar and Martian gravities either grew just as fast as simulated microgravity, or somewhere in between simulated microgravity and 1 g. The 4157 strain also showed a faster growth rate under the simulated reduced gravities with respect to 1 g, but in this case, simulated lunar gravity was the fastest growing (14% with respect to 1 g). Comparing these results with spaceflight data shows that these increases in exponential growth rate do not agree with the EcAMSat spaceflight mission, which found that the growth rate of AMG1 and ∆*rpoS* strains was unaltered by the microgravity environment [40], although it should be noted that it was acknowledged that their cells may have been under stress from the culturing hardware, and that different media were used in the two experiments. Similarly, Klaus et al. [34] found that the rate of growth for cells grown in microgravity was ‘unaffected (or slightly slower)’, again contrary to our results, albeit under different experimental conditions. The exposure to cell byproducts and waste described in the latter paragraph has also been discussed to moderate growth during the exponential phase for *E. coli* 4157 [20,34,50]. This model suggests that nonmotile cells may experience a decrease in exponential growth because of an altered metabolism due to a lack of sedimentation. The results of cultures grown under simulated microgravity do not support this theory, as the nonmotile *E.coli* 4157 strain experienced a slightly higher exponential growth rate in simulated microgravity than the 1 g control. Meanwhile, the sedimentation that occurred in simulated lunar gravity was potentially sufficient to allow cells to ‘escape’ byproducts and have access to more nutrients, thus yielding the highest observed growth rate when compared to the 1 g control for this strain.

In our study, the three strains reached the stationary phase by 12 h. The AMG1 and 4157 strains showed no significant differences in OD as a function of gravitational condition at this time. On the other hand, the ∆*rpoS* strain had the highest OD under simulated microgravity (35% increase with respect to 1 g), the lowest at 1 g, and sLg and sMg in between. At experiment end (t = 24 h), the highest growth was observed under simulated microgravity on the AMG1 strain (48% increase with respect to 1 g) (with simulated lunar and Martian gravities in between), and under simulated micro and Lunar gravities on the ∆*rpoS* strain (44% increase with respect to 1 g) (with simulated Martian gravity in between). The 4157 strain showed the opposite: the highest growth was at 1 g, and the lowest under simulated microgravity (17% decrease with respect to 1 g). In this case, and keeping in mind the caveat regarding different media used for 4157, a correlation was observed that the non-motile strain had less growth in simulated microgravity and most in 1 g, while the opposite was true for the semi-motile and motile strains. The observed higher yield in simulated microgravity with respect to 1 g of the 4157 strain also contradicts observations made on the same flight hardware (albeit in minimal media) in actual microgravity, where a 13 fold increase was observed in the spaceflight samples with respect to Earth controls [7]. If the increase in growth under simulated micro- and lunar gravities of the uropathogenic strains (AMG1 and AMG1 ∆*rpoS*) holds true in these environments, this may have a negative impact by increasing the likelihood of crewmembers developing UTIs, a problem frequently reported by astronauts [51].

Cellular size measurements, taken from samples fixed at experiment end (t = 24 h), showed that AMG1 cells grown under simulated microgravity were 17% longer and 11% wider with respect to 1 g controls. In the case of the ∆*rpoS* strain, the only difference in cell size observed was a 6% increase in length with respect to 1 g on the cells cultured under simulated Martian gravities. No statistical differences were observed on *E.coli* 4157 cell length or diameter as a function of gravitational condition. Our overall cell size observations are not in agreement with our previous spaceflight results, where *E. coli* 4157 cells cultured in space were 59% the length, and 83% the diameter, of their Earth controls [21]. In terms of aggregation, none was observed in the AMG1 and ∆*rpoS* strains, and it was present in 4157 cultures, but exclusively those cultured under simulated microgravity. This is in agreement with our previous 4157 study, where cellular aggregation was observed in spaceflight samples fixed in PFA upon return to Earth, while none was seen in the Earth controls [21].

Regarding the MICs, the uropathogenic AMG1 strain showed that lower concentrations of Ciprofloxacin, the most commonly used antibiotic against UTIs on Earth [43], were needed under the three simulated reduced gravities with respect to 1 g, despite the fact that these gravitational conditions yielded higher cell counts in the growth dynamics study. Significantly, it was observed that cultures grown in simulated lunar gravity needed twice the concentration of simulated micro and Martian gravities, but still less than 1 g. Similarly, the ∆*rpoS* strain needed a lower concentration of antibiotics to inhibit growth under simulated microgravity with respect to the other three gravitational conditions. Finally, for the 4157 strain, the simulated lunar gravity cultures required the highest concentration of Gentamicin for growth to be inhibited: 18 mg/L, compared to 12 mg/L needed for the other three gravitational conditions.

Hence, this study showed differences in growth dynamics, cellular size, and aggregation as a function of time and *E. coli* strain under clinorotation, results that may be verified and further studied under axial centrifugation in microgravity, in order to more realistically achieve simulated lunar and Martian gravities. Nevertheless, our inclined clinostat and in-house code methodology may enable preliminary partial gravity studies to be performed on Earth by other teams, with the caveat that this methodology (just like all reduced gravity simulation devices including clinostats, rotating wall vessels, and random positioning machines) still need to go through a thorough verification and validation process using spaceflight assets.

## Figures and Tables

**Table 1:** Slopes of each gravitational regime between the first and last points of the exponential phase for each strain. R^2^ > 0.90 and >0.94 for all slopes of AMG1 and AMG1 ∆rpoS, respectively; wrt = with respect to.

Gravitational Regime	*E. coli* AMG1		*E. coli* AMG1 ∆*rpoS*	
	Slope	Slope wrt 1 g	Slope	Slope wrt 1 g
Simulated microgravity	0.048	117%	0.079	123%
Simulated lunar	0.045	111%	0.078	120%
Simulated Martian	0.048	117%	0.071	111%
1 g	0.041	N/A	0.065	N/A

**Table 2:** Death rate slopes (between the last two timepoints) of each gravitational regime; wrt = with respect to.

Gravitational Regime	*E. coli* AMG1		*E. coli* AMG1 ∆*rpoS*	
	Slope	Slope wrt 1 g	Slope	Slope wrt 1 g
Simulated microgravity	−0.0009	26%	−0.0036	112%
Simulated lunar	−0.0031	91%	−0.0023	72%
Simulated Martian	−0.0039	115%	−0.0025	78%
1 g	−0.0034	N/A	−0.0032	N/A

**Table 3:** Average cell sizes per gravitational condition for each strain (*n* = 50). Length and diameter values in µm. *: 0.01 < *p* ≤ 0.05; ***: *p* ≤ 0.001; wrt = with respect to; sµm, sLg, sMg: simulated micro, lunar, and Martian gravity, respectively.

Gravitational Regime	AMG1 Length	∆*rpoS* Length	∆*rpoS* Length wrt AMG1	AMG1 Length wrt 1 g	∆*rpoS* Length	AMG1 Width	∆*rpoS* Width	∆*rpoS* Width wrt AMG1	AMG1 Width wrt 1 g	∆*rpoS* Width wrt 1 g
sµg	3.00	2.37	79%	117% ***	106%	0.95	0.94	99%	111% ***	104%
sLg	2.98	2.36	79%	117% ***	105%	0.86	0.94	110%	100%	104%
sMg	2.75	2.37	86%	107% *	106% *	0.87	0.90	103%	101%	100%
1 g	2.56	2.24	88%	N/A	N/A	0.86	0.90	105%	N/A	N/A

**Table 4:** Identified Minimum Inhibitory Concentration (MIC) (mg/L) of Ciprofloxacin as a function of gravitational condition and *E. coli* strain; > in the table means that the MIC was greater than the highest concentration of antibiotic tested (in this case, 0.50).

Gravitational Regime	*E. coli* AMG1	*E. coli* AMG1 ∆*rpoS*
Simulated microgravity	0.25	0.50
Simulated lunar	0.50	>0.50
Simulated Martian	0.25	>0.50
1 g	>0.50	>0.50

**Table 5:** Slopes of each gravitational regime between 1.5 and 6 h.

Gravitational Regime	Slope	Slope wrt 1 g
Simulated microgravity	0.044	105%
Simulated lunar	0.047	114%
Simulated Martian	0.045	108%
1 g	0.042	N/A

**Table 6:** **MIC of Gentamicin Sulfate.** Identified Minimum Inhibitory Concentration (MIC) (mg/L) of Gentamicin Sulfate on *E. coli* 4157 as a function of gravitational condition.

Gravitational Regime	*E. coli* 4157
Simulated microgravity	12
Simulated lunar	18
Simulated Martian	12
1 g	12

## Data Availability

The code presented in this study is openly available in GitHub at https://github.com/SpaceLuisZea/Clinostat (accessed on 9 August 2022).

## Nomenclature

RPM	revolution per minute
MIC	minimum inhibitory concentration
UTI	urinary tract infection
ISS	International Space Station
NASA	National Aeronautics and Space Administration
FPA	fluid processing apparatus
sµg	simulated microgravity
sLg	simulated Lunar gravity
sMg	simulated Martian gravity
mAUMg	hi-Pi modified artificial urine medium (high concentration of phosphate)
OD	optical density
PFA	paraformaldehyde
ANOVA	Analysis of Variance
Nm	nanometer
µm	micrometer
wrt	with respect to
EcAMSat	Esherichia coli Antimicrobial Satellite
